# Colonic pleomorphic undifferentiated sarcoma with a leukemoid reaction: case report

**DOI:** 10.3389/fmed.2026.1883302

**Published:** 2026-07-01

**Authors:** Ruizhi Wang, Tao Shan, Dong Mu, Jiayi Cheng, Baoguang Hu, Fengjie Wu

**Affiliations:** 1 Department of Gastrointestinal Surgery, Binzhou Medical University Hospital, Binzhou, China; 2 Department of General Surgery, Bincheng District People's Hospital, Binzhou, China

**Keywords:** case report, colon, leukemoid reaction, malignant fibrous histiocytoma, undifferentiated pleomorphic sarcoma

## Abstract

**Background:**

Undifferentiated pleomorphic sarcoma (UPS) is an aggressive mesenchymal tumor that typically occurs in the extremities or retroperitoneum. Primary colonic involvement is relatively rare, and those accompanied by a leukemoid reaction are even rarer. This case of primary colonic UPS with a significant leukemoid reaction highlights the unique nature of this rare paraneoplastic syndrome, which may mimic hematologic malignancy and delay appropriate surgical intervention. It also contributes new clinical evidence to the literature by indicating that surgical reduction of tumor burden can reverse tumor-associated leukocytosis and support a paraneoplastic mechanism.

**Case description:**

A 49-year-old woman presented with a 3-month history of fatigue, poor appetite, and intermittent low-grade fevers. Laboratory evaluation revealed severe anemia (Hb 56 g/L) and extreme leukocytosis (WBC 41.1 × 10^9^/L) with a left shift, indicating a leukemoid reaction. Bone marrow aspiration and flow cytometry excluded hematologic malignancy, showing only reactive granulocytic hyperplasia. Colonoscopy identified a large, irregular, obstructing mass at the splenic flexure. Contrast-enhanced CT confirmed a colonic tumor with local invasion. After multidisciplinary discussion, the patient underwent radical multivisceral resection, which included left hemicolectomy, distal pancreatectomy, splenectomy, and hysterectomy with bilateral salpingo-oophorectomy. Pathological examination confirmed undifferentiated pleomorphic sarcoma. The proximal and distal colonic margins were negative, whereas sarcomatous tissue was found on the pancreatic tail resection margin. Postoperatively, the leukocyte counts rapidly normalized, and the leukemoid reaction resolved completely. The patient had an uneventful postoperative recovery, and the patient was discharged without early complications.

**Conclusion:**

This case underscores that primary colonic UPS can present with a paraneoplastic leukemoid reaction, which may be mistaken for a primary hematologic disorder. When hematologic malignancy is excluded, such a reaction should prompt evaluation for an underlying solid tumor. Multivisceral resection can reduce tumor burden and may reverse paraneoplastic leukocytosis, while margin status remains critical for postoperative management and long-term surveillance. Multidisciplinary collaboration is required for accurate diagnosis and optimal management of these rare and aggressive tumors.

## Introduction

UPS is an aggressive mesenchymal tumor typically found in the extremities or retroperitoneum. Primary colonic involvement is exceptionally rare. Because the symptoms are non-specific, the diagnosis usually occurs late—when the tumor grows large or complications develop (1). UPS is formed from mesenchymal cells and has significant cellular heterogeneity and nuclear pleomorphism on pathology, with immunohistochemistry confirming mesenchymal origin. The exact causes are unknown, but genetic factors, chronic inflammation, or persistent irritants may play a role (2, 3).

The prognosis is poor. UPS recurs locally and spreads frequently, making management difficult. Primary colorectal UPS is even rarer (1, 4). Because these tumors form deep within the intestinal wall and have no early symptoms, they are frequently misdiagnosed as colorectal carcinoma or inflammatory bowel disease. Most published reports are single cases or small series; no comprehensive review exists.

A leukemoid reaction is defined as a significant increase in the peripheral white blood cell count in the absence of leukemia. Diagnostic thresholds vary, but often accept WBC > 40,000–50,000/μL with a left shift and no leukemic clone. Severe infections, tumor-associated inflammation, and malignancies are also possible causes (5, 6).

UPS with leukemoid reaction is exceedingly rare. Primary colonic UPS with significant leukocytosis is even rarer. Moreover, few reports have documented postoperative normalization of leukocytosis, suggesting a paraneoplastic mechanism. This case therefore addresses an important gap in the literature by describing the diagnostic workup, pathological findings, postoperative leukocyte response, and clinical implications of this unusual presentation. We reported a rare case of primary colonic UPS presenting as an obstructing colonic mass with significant leukocytosis, in which the leukocyte count rapidly normalized after multivisceral resection. We also review previously reported cases of colorectal UPS to contextualize the clinicopathological features, treatment strategies, and outcomes of this rare disease.

## Case presentation

A 49-year-old woman presented to our hospital reporting fatigue for over 3 months and a high white blood cell count. Three months before admission, she had fatigue no evident cause, as well as a poor appetite and intermittent low fevers. No significant abdominal pain, melena, or vomiting. Tests at an outside hospital showed persistently high WBC and severe anemia. Antibiotics did not work, so she came to us.

History: hypertension for 1 year, well controlled with oral medication. Uterine fibroid surgery was performed 25 years ago.

Exam: On admission, the patient appeared pale and had conjunctival pallor. Her body temperature was 36.6 °C, blood pressure was 123/75 mmHg, heart rate was 127 beats/min, and respiration rate was 20 breaths/min. No superficial lymphadenopathy was palpable. Cardiopulmonary examination was unremarkable except for tachycardia. The abdomen was soft, without tenderness, rebound tenderness, guarding, or palpable mass. There were no symptoms of peritoneal irritation or obvious skin infection.

Lab results: WBC 41.1 × 10^9^/L, RBC 2.8 × 10^12^/L, Hb 56 g/L, and platelets 428 × 10^9^/L. Differential: lymphocytes 6.3%, neutrophils 85.7%, monocytes 1.6 × 10^9^/L, neutrophils 35.2 × 10^9^/L, eosinophils 1.48 × 10^9^/L, basophils 0.15 × 10^9^/L. CRP 141 mg/L, and CA125 111.00 U/mL. The WBC count remained markedly elevated despite antibiotic therapy (Figure 1). Bone marrow aspiration and flow cytometry showed reactive hyperplasia with granulocytic proliferation but no blasts or abnormal clones, ruling out hematologic malignancy.

**Figure 1:**
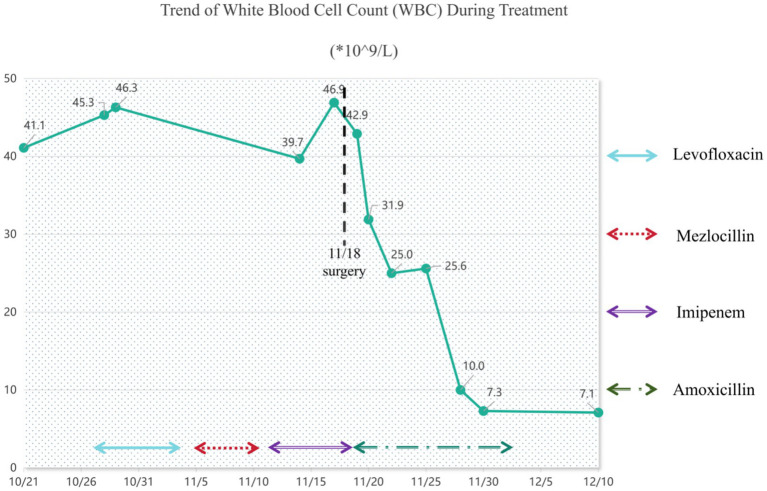
Trend of the white blood cell (WBC) count during treatment. Despite antibiotic therapy, the WBC count remained significantly elevated before surgery. After multivisceral tumor resection on November 18, the WBC count gradually decreased and returned to the normal range. The arrows indicate the duration of antibiotic treatment.

Colonoscopy found a large irregular mass at the splenic flexure that encircled the lumen. The surface had deteriorated and bled easily, and the colonoscope could not be advanced beyond the lesion (Figure 2). The contrast-enhanced abdominal CT showed a splenic flexure mass with indistinct margins, suggesting advanced transverse colon cancer with narrowing (Figure 3).

**Figure 2:**
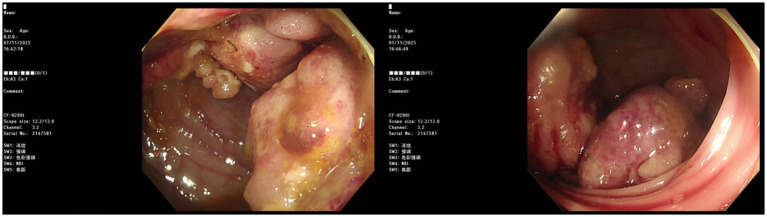
Colonoscopy found a large, irregular mass at the splenic flexure. Colonoscopy findings: at the splenic flexure, an irregular, nodular, large lesion involving the entire circumference of the colon was observed. The surface was covered with exudate, and the lesion was friable and bled easily.

**Figure 3:**
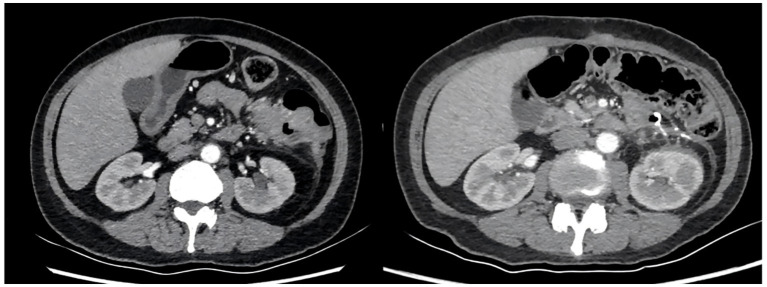
Contrast-enhanced abdominal CT showed a splenic flexure mass with indistinct margins (preoperative and postoperative). Contrast-enhanced abdominal CT revealed patchy abnormal density at the splenic flexure of the colon, with a maximum cross-sectional area of approximately 5.6 cm × 6.2 cm. The density was heterogeneous, with internal calcifications and mildly enlarged lymph nodes. There was no significant enhancement on contrast-enhanced scan. Contrast-enhanced CT of the abdomen and pelvis shows multiple linear and patchy hyperdense opacities in the anterior abdominal wall, consistent with postoperative changes. The surgical bed shows linear dense opacities and small cystic/patchy hypodense areas, as well as the partial disappearance of the left colon, pancreatic tail, and spleen, which are consistent with postoperative changes. Fat planes in the surgical bed are unclear, with linear and patchy hyperdense opacities and ill-defined margins. There is no bowel wall thickening or luminal dilation. There is no retroperitoneal lymphadenopathy. There is no pelvic fluid collection or enlarged lymph nodes.

Given persistent extreme leukocytosis, bone marrow findings, and colonic mass, we considered colonic malignancy with leukemoid reaction. After multidisciplinary discussion, anemia was treated preoperatively, and surgical treatment was performed (Table 1).

**Table 1:** Timeline of diagnosis, treatment, and response.

Date	Clinical manifestations/Symptoms	Key investigations	Main interventions/clinical decisions
21 October 2025	Fatigue, poor appetite, diarrhea, subjective low-grade fever, and transient high fever.	Complete blood count: leukocytosis (41.1*10^9^), anemia	Admitted to the Department of Hematology; Red blood cell transfusion
23 October 2025–27 October 2025	Still fatigued, anemic appearance, pale conjunctiva and lips, coarse breath sounds in both lungs.	Bone marrow aspirate/biopsy: ruled out acute leukemia. Abdominal US: gallbladder polyps, splenomegaly, left upper quadrant mixed echogenicity (possible bowel origin). Contrast CT: splenic flexure mass (5.6 × 6.2 cm), calcifications, regional lymphadenopathy; colonoscopy advised.	Transferred to the Department of Gastrointestinal Surgery. Empirically administered levofloxacin for anti-infective therapy.
28 October 2025–17 November 2025	anemia, recurrent fever, leukocytosis	Colonoscopy: Transverse colon cancer (advanced) with stenosis. Complete blood count: RBC 3.0*10^12^/L, WBC 48.3*10^9^/L, hemoglobin 71 g/L, and procalcitonin 0.284 ng/mL. Blood culture: *E. coli*.	Suspected leukemoid reaction; allogeneic blood transfusion administered; started on mezlocillin for anti-infective therapy, then switched to imipenem based on culture and susceptibility results.
18 November 2025	Surgical treatment	Surgical treatment	Left hemicolectomy, distal pancreatectomy, splenectomy, transabdominal extrafascial total hysterectomy with bilateral adnexectomy, and sacrospinous ligament suspension of the vaginal cuff.
19 November 2025–28 November 2025	Postoperative general condition fair; incisional pain; occasional fever; abdominal drain output approximately 50 mL/day.	White blood cell counts gradually decreased (42.9–10.0*10^9^/L); platelet count: 1175*10^9^/L.	Considering reactive thrombocytosis after splenectomy, administered aspirin and dipyridamole; amoxicillin for anti-infective therapy.
29 November 2025–19 December 2025	Stable condition	Platelet count decreased; white blood cell count returned to normal level.	Received regular dressing changes; recovered well; discharged from hospital.
12 March 2026	Admitted to the Department of Oncology	Platelet count: 1061*10^9^/L, hemoglobin 61 g/L, contrast-enhanced CT of the abdomen and pelvis: no bowel wall thickening, no enlarged lymph nodes or space-occupying lesions.	After blood transfusion, the family requested transfer to a higher-level hospital.

During surgery: a tumor at the splenic flexure, approximately 8 cm × 6 cm, had penetrated serosa and invaded the pancreatic tail and splenic hilum. Multiple nodules in the uterus and adnexa. We performed a left hemicolectomy with distal pancreatectomy, splenectomy, and hysterectomy with bilateral adnexectomy.

Pathology confirmed undifferentiated pleomorphic sarcoma. The proximal and distal colonic margins were negative; however, sarcomatous tissue was found in the pancreatic tail parenchyma, which included the pancreatic tail resection margin. The patient had an uneventful postoperative recovery. The WBC count gradually normalized, and the leukemoid reaction resolved.

### Pathological findings

Left hemicolon: Sarcoma consistent with undifferentiated pleomorphic sarcoma involving pericolonic fat beneath serosa. No neural invasion or vascular tumor thrombi. Margins clear. No metastatic tumor in pericolonic lymph nodes (0/6). Additional nodules contained sarcomatous tissue. Pancreatic tail: Sarcomatous tissue in parenchyma, involving resection margin. Spleen: No tumor. Uterus and adnexa: Multiple leiomyomas. Endometrium proliferative phase, chronic cervicitis. Bilateral ovarian serous cystadenomas. Fallopian tubes unremarkable. Pelvic cavity: Leiomyoma. Immunohistochemistry: Vimentin and CD68 positive; SDHB and *β*-catenin weakly positive; CK, CD117, DOG-1, CD34, S-100, SMA, Desmin, Caldesmon, and ALK negative; Ki-67 ~ 20% (Figure 4).

**Figure 4:**
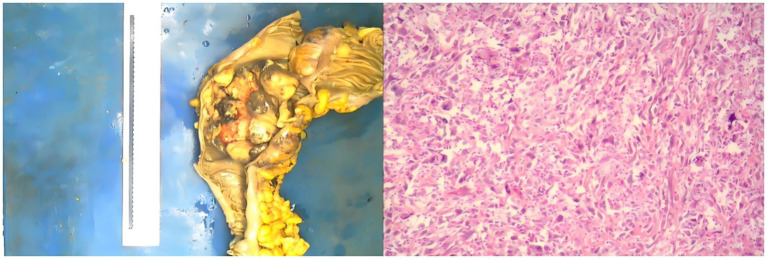
Pathological examination. Left hemicolon: sarcoma consistent with undifferentiated pleomorphic sarcoma. Gross examination revealed a large circumferential exophytic tumor in the left hemicolon, measuring 9 × 4.5 × 3 cm, with a gray-white to gray-yellow soft cut surface and extension toward the pericolonic adipose tissue. Microscopic examination showed markedly pleomorphic spindle and epithelioid tumor cells with prominent nuclear atypia, supporting the diagnosis of undifferentiated pleomorphic sarcoma (H&E, ×100).

### Follow-up and outcomes

At the latest documented follow-up, on 12 March 2026, the patient was admitted to the Department of Oncology for further evaluation. Laboratory tests showed marked thrombocytosis, with a platelet count of 1,061 × 10^9^/L, and severe anemia with a hemoglobin level of 61 g/L. A contrast-enhanced CT scan of the abdomen and pelvis showed no bowel wall thickening, enlarged lymph nodes, or space-occupying lesions, and no radiological evidence of local recurrence or distant metastasis was identified at that time. After a blood transfusion, the patient’s family requested a transfer to a higher-level hospital. Further follow-up information after transfer, including as subsequent adjuvant therapy and long-term oncological outcomes, was unavailable.

## Discussion

Ozzello et al. first described undifferentiated pleomorphic sarcoma (UPS) in 1963, which was previously classified as malignant fibrous histiocytoma (MFH). In 2002, the World Health Organization revised the classification of soft tissue tumors, reassigning several subtypes previously categorized as MFH. The storiform-pleomorphic, inflammatory, and giant cell variants were unified under the term UPS. This reclassification underscored the fundamental nature of UPS as a highly malignant mesenchymal neoplasm lacking a definite line of differentiation (7).

To further elucidate the clinical characteristics and prognostic features of colorectal UPS, we conducted a systematic search of the PubMed database up to 1 January 2026. The inclusion criteria were as follows: (1) cases with complete or accessible clinical data, (2) tumors originating in the colon or rectum and confirmed as UPS by histopathological and immunohistochemical examination, and (3) study type limited to case reports. Exclusion criteria included duplicate case reports, tumors not primarily arising from the colorectum, and studies unrelated to UPS. After screening, a total of 29 cases of colorectal UPS meeting the criteria were identified, and the relevant data are summarized in Table 2. Based on these cases, we performed a systematic analysis of the clinical features of colorectal UPS.

**Table 2:** UPS of colorectum in the literature.

Author (Reference No.)	Age (years)	Sex	Tumor site	Maximum diameter (cm)	Symptoms	Surgery	Adjuvant therapy	Follow-up and outcome
Verma P et al. (1979) (12)	38	Male	Rectum	12	Abdominal pain	Yes	NA	14 months: NED
Sewell et al. (1980) (13)	74	Male	Transverse colon	8.5	Anorexia; diarrhea	Yes	None	12 months: no recurrence or metastasis
Levinson & Tsang (1982) (14)	17	Male	Transverse colon + sigmoid colon	10; 8	Abdominal pain	Yes	NA	NA
Rubbini et al. (1983) (15)	60	Male	Sigmoid colon	7	Hematochezia	Yes	Chemotherapy	53 months: died; liver metastasis
Waxman et al. (1983) (16)	52	Female	Sigmoid colon	7.5	Abdominal pain	Yes	None	9 months: died; local recurrence
Spagnoli L.G. (1984) (17)	52	Female	Anorectum (anorectal region)	2.9	Hematochezia	Yes	None	24 months: died; local recurrence and lung metastasis
Baratz et al. (1986) (18)	73	Male	Transverse colon	15	Anorexia; anemia	Yes	None	6 months: no recurrence or metastasis
Satake & Matsuyama (1988) (19)	62	Male	Ascending colon + transverse colon	17; 19	Abdominal mass	No	NA	NA
Flood H.D. (1989) (20)	41	Male	Anal canal	6	Perianal pain	Yes	Radiotherapy	16 months: NED
Katz et al. (1990) (21)	62	Female	Cecum	2	Abdominal pain	Yes	None	3 months: no recurrence or metastasis
Huang & Wei (1993) (22)	12	Male	Ascending colon	3.5	Abdominal pain	Yes	None	16 months: no recurrence or metastasis
Murata et al. (1993) (23)	50	Male	Ascending colon	9.5	Abdominal distension	Yes	Chemotherapy	10 months: no recurrence or metastasis
Makino et al. (1994) (24)	72	Male	Transverse colon	7	Abdominal pain	Yes	None	4 months: died; local recurrence
Hiraoka et al. (1997) (25)	64	Male	Cecum	5	Abdominal distension	Yes	None	4 months: died; lymph node metastasis
Kawashima et al. (1997) (26)	50	Female	Descending colon	10	Abdominal pain	Yes	None	7 years: no recurrence or metastasis
Singh et al. (1999) (27)	55	Male	Rectum	2.5	Perineal pain	Yes	Chemotherapy + radiotherapy	46 months: no recurrence or metastasis
Udaka et al. (1999) (28)	47	Male	Ascending colon	7	Abdominal mass	Yes	None	13 months: no recurrence or metastasis
Okubo et al. (2005) (29)	66	Male	Ascending colon	14.5	Abdominal pain	Yes	None	33 months: no recurrence or metastasis
Gupta & Malani (2006) (30)	46	Female	Cecum + ascending colon	17	Abdominal distension; abdominal mass	Yes	None	36 months: no recurrence or metastasis
Bosmans et al. (2007) (31)	73	Male	Sigmoid colon	3.5	Anemia	Yes	None	22 months: no recurrence or metastasis
Fu DL et al. (2007) (32)	70	Male	Cecum	12	Abdominal pain	Yes	None	1 month: died; lung metastasis
Kim BG et al. (2008) (33)	63	Female	Anal canal	1.7	Hematochezia	Yes	Radiotherapy	15 months: NED
Azizi R et al. (2011) (34)	80	Male	Rectum	5	Rectal bleeding	Yes	None	NA
Wang et al. (2012) (35)	55	Male	Sigmoid colon	6.0	Abdominal pain	Yes	None	5 months: died; local recurrence
Ji et al. (2016) (36)	68	Female	Ascending colon	8	Fever	Yes	Radiotherapy	5 years: died; local recurrence
Han et al. (2022) (7)	65	Female	Descending colon	10	Fever; hematochezia; abdominal distension	Yes	None	12 months: NED
Kodera et al. (2022) (rectal UPS) (37)	85	Female	Rectum	7.5	Anal pain	Yes	Radiotherapy	16 months: NED
McCombs D et al. (2023) (38)	12	Female	Cecum	7	Nausea, vomiting, abdominal pain	Yes	None	12 months: NED
Qureshi PAAA et al. (2024) (39)	49	Female	Descending colon	8	Fever; abdominal pain	Yes	None	36 months: NED

NA, not available; NED, no evidence of disease.

Since 1979, only a limited number of colorectal UPS/MFH cases have been reported, mostly as isolated case reports. In our review of 29 reported colorectal UPS cases, the disease showed a slight male predominance, with 18 male and 11 female patients, and the mean age at diagnosis was 55.62 ± 18.49 years. Tumors most commonly appeared in the right colon, then the left colon and rectal or anorectal region. Most tumors were large at presentation, with a mean maximum diameter of 8.09 ± 4.40 cm, indicating non-specific clinical manifestations and delayed diagnosis of this rare tumor. The most common presenting symptom was abdominal pain, followed by gastrointestinal bleeding, abdominal distension, palpable mass, fever, and perineal or perianal pain. Most patients underwent surgical resection, but adjuvant therapy varied significantly. During follow-up, recurrence or metastasis was reported in 8 of 26 patients with available outcome data, highlighting the aggressive biological behavior of colorectal UPS (Table 2).

Because colorectal UPS may develop from deep mesenchymal tissues in the intestinal wall, the intestinal mucosa may remain intact early, resulting in missed lesions on colonoscopy. The definitive diagnosis of UPS is based on histopathological and immunohistochemical examinations. Histologically, UPS is characterized by marked cellular pleomorphism, pronounced nuclear atypia, tumor necrosis, and a high mitotic index. Immunohistochemically, tumor cells typically express vimentin, CD68, α1-antitrypsin, or α1-antichymotrypsin, but lack lineage-specific differentiation markers. The main role of immunohistochemistry is to exclude other sarcomas, carcinoma, lymphoma, melanoma, and other mimics. In this case, negative immunohistochemical staining for CD117, DOG-1, and CD34 did not support a diagnosis of gastrointestinal stromal tumor; negative staining for SMA, desmin, and caldesmon did not support leiomyosarcoma; negative staining for CK did not support sarcomatoid carcinoma; negative staining for S-100 did not support melanoma or malignant peripheral nerve sheath tumor; and negative staining for ALK did not support inflammatory myofibroblastic tumor. Dedifferentiated liposarcoma was also considered, but no well-differentiated liposarcomatous component or lipoblasts were identified. Weak SDHB and *β*-catenin staining was observed; however, these findings were not considered diagnostically defining in this case. Weak SDHB staining was not interpreted as loss of SDHB expression and did not support a specific SDH-deficient tumor. Similarly, weak β-catenin staining, in the absence of convincing diffuse nuclear positivity, was interpreted as non-specific and did not support desmoid-type fibromatosis. Overall, the pleomorphic spindle cell morphology, vimentin/CD68 positivity, and absence of lineage-specific differentiation supported the diagnosis of UPS.

A leukemoid reaction represents a reactive condition characterized by extreme leukocytosis and is commonly associated with infection, inflammation, or malignancy. Tumor-related leukocytosis may result from tumor-derived cytokines, such as granulocyte colony-stimulating factor (G-CSF), which stimulate bone marrow proliferation. Previous reports have suggested that UPS and other sarcomas may secrete G-CSF, thereby causing systemic inflammatory responses and marked leukocytosis (8). Leukemoid reactions associated with malignant tumors may carry serious implications. Abukhiran et al. reviewed leukemoid reactions in various solid tumors and found they correlate with tumor aggressiveness and poor prognosis (6).

Our patient had a leukemoid reaction but no evidence of hematologic malignancy on bone marrow examination or flow cytometry. Infection is an important differential diagnosis in patients with extreme leukocytosis. In the present case, the preoperative blood culture grew *Escherichia coli*, indicating that infection may have contributed to the elevated WBC count. However, the leukocytosis persisted despite antibiotic therapy and rapidly resolved after tumor resection. Therefore, the leukocytosis in this case was likely multifactorial, with documented *E. coli* bacteremia as a contributing factor and tumor-associated paraneoplastic stimulation as a substantial component. The postoperative hematologic response suggests that tumor burden may have remained a key driver of the leukemoid reaction. Nevertheless, the coexistence of documented bacteremia and suspected paraneoplastic leukemoid reaction makes it difficult to precisely determine the relative contribution of infection and tumor burden, which represents an important limitation of this case.

### Therapeutic strategies and prognosis

Complete surgical resection remains key for UPS. For tumors with extensive local invasion, a multimodal approach incorporating surgery, radiotherapy, and chemotherapy should be considered to improve local disease control (9–11). At present, no consensus exists on routine adjuvant radiotherapy or chemotherapy after surgery. Decisions should be individualized based on pathological subtype, tumor stage, and overall disease burden.

Colonic sarcoma with leukemoid reaction and multiorgan involvement may appear clinically aggressive, but timely diagnosis and individualized multidisciplinary management may still provide meaningful disease control. Clinicians should recognize this entity to avoid mistaking it for a primary hematologic disorder based solely on high WBC, allowing earlier diagnosis and precise treatment. In our case, preoperative antibiotics did not reduce the WBC count, whereas multivisceral tumor resection was followed by rapid leukocyte normalization. This suggests tumor burden drives the reaction and supports prompt surgery once diagnosed. Multidisciplinary teamwork is essential for individualized care. Symptom management, surgical reduction of tumor burden, and individualized postoperative treatment may improve disease control. After surgery, focus on adjuvant therapy and long-term follow-up with regular CBC, tumor markers, and imaging to catch recurrence early.

## Conclusion

Primary colonic UPS is extremely rare. Cases with leukemoid reaction are even rarer, rendering them highly susceptible to misdiagnosis as hematologic malignancies. This case reports a colonic UPS presenting initially with a marked leukemoid reaction unresponsive to antibiotics. After multivisceral tumor resection, WBC quickly normalized, demonstrating that when hematologic disease is ruled out, leukemoid reaction should prompt evaluation for curative resection, which can reverse the paraneoplastic state and improve quality of life and prognosis. This case highlights that multivisceral tumor resection not only provides a definitive diagnosis but also effectively resolves the leukemoid reaction, offering insights for managing such complex cases.

## Patient perspective

The patient and her family reported significant concern during the diagnostic process because of the persistent leukocytosis, severe anemia, and uncertainty regarding the underlying disease. After surgery, they understood the suspected tumor-associated nature of the leukemoid reaction and agreed with the need for further oncologic evaluation and close follow-up.

## Data Availability

The original contributions presented in the study are included in the article/supplementary material, further inquiries can be directed to the corresponding authors.

## References

[1] Lu Y Chen D Wang B Chai W Yan M Chen Y . Single-cell landscape of undifferentiated pleomorphic sarcoma. Oncogene. (2024) 43:1353–68. doi: 10.1038/s41388-024-03001-8, 38459120

[2] Koelsche C Benhamida JK Kommoss FKF Stichel D Jones DTW Pfister SM . Intimal sarcomas and undifferentiated cardiac sarcomas carry mutually exclusive MDM2, MDM4, and CDK6 amplifications and share a common DNA methylation signature. Mod Pathol. (2021) 34:2122–9. doi: 10.1038/s41379-021-00874-y, 34312479 PMC8592836

[3] Tvrdik T Gjeorgjievski SG Wong P Oskouei S Read W Bahrami A. Genomic insights into high-grade infarct-associated bone sarcomas. Mod Pathol. (2024) 37:100572. doi: 10.1016/j.modpat.2024.100572, 39033963

[4] Wu F Feng J Wang H Wang S Cui X Liu Y . Efficacy of toripalimab in combination with anlotinib in recurrent undifferentiated pleomorphic sarcoma of the sinonasal region: a case report with biomarker analysis. Front Immunol. (2025) 16:1541209. doi: 10.3389/fimmu.2025.1541209, 40416963 PMC12098334

[5] Iyengar V Meyer A Stedman E Casale S Kalsi S Hale AJ . A predictive algorithm for discriminating myeloid malignancies and Leukemoid reactions. Am J Med. (2024) 137:658–65. doi: 10.1016/j.amjmed.2024.03.015, 38499135

[6] Abukhiran I Mott SL Bellizzi AM Boukhar SA. Paraneoplastic leukemoid reaction: case report and review of the literature. Pathol Res Pract. (2021) 217:153295. doi: 10.1016/j.prp.2020.153295, 33341546

[7] Han X Zhao L Mu Y Liu G Zhao G He H . Undifferentiated high-grade pleomorphic sarcoma of the colon: a rare case report and literature review. BMC Gastroenterol. (2022) 22:115. doi: 10.1186/s12876-022-02189-x, 35272624 PMC8908612

[8] Hayashida K Kawabata Y Kato I Suzuki M Takeyama M Inaba Y. G-CSF production by undifferentiated pleomorphic sarcoma with Leukemoid reaction occurred in the lower leg: a case report. JBJS Case Connect. (2020) 10:e0624. doi: 10.2106/jbjs.Cc.19.00624, 32649136

[9] van Oost S Meijer DM Erdem ZB IJsselsteijn ME Roelands J Lam SW . Divergent therapeutic and prognostic impacts of immunogenic features in undifferentiated pleomorphic sarcoma and myxofibrosarcoma. Cancer Immunol Immunother. (2025) 74:258. doi: 10.1007/s00262-025-04123-y, 40601026 PMC12222583

[10] Jibbe A Worley B Miller CH Alam M. Surgical excision margins for fibrohistiocytic tumors, including atypical fibroxanthoma and undifferentiated pleomorphic sarcoma: a probability model based on a systematic review. J Am Acad Dermatol. (2022) 87:833–40. doi: 10.1016/j.jaad.2021.09.036, 34587553

[11] Danieli M Barretta F Radaelli S Fiore M Sangalli C Barisella M . Pathological and radiological response following neoadjuvant treatments in primary localized resectable myxofibrosarcoma and undifferentiated pleomorphic sarcoma of the extremities and trunk wall. Cancer. (2023) 129:3417–29. doi: 10.1002/cncr.34945, 37452607

[12] Verma P Chandra U Bhatia PS. Malignant histiocytoma of the rectum: report of a case. Dis Colon Rectum. (1979) 22:179–82. doi: 10.1007/bf02586815, 221161

[13] Sewell R Levine BA Harrison GK Tio F Schwesinger WH. Primary malignant fibrous histiocytoma of the intestine: intussusception of a rare neoplasm. Dis Colon Rectum. (1980) 23:198–201. doi: 10.1007/bf02587627, 6247126

[14] Levinson MM Tsang D. Multicentric malignant fibrous histiocytomas of the colon. Report of a case and review of the subject. Dis Colon Rectum. (1982) 25:327–31. doi: 10.1007/bf02553607, 6282547

[15] Rubbini M Marzola A Spanedda R Scalco GB Zamboni P Guerrera C . Primary malignant fibrous histiocytoma of the sigmoid colon: a case report. Ital J Surg Sci. (1983) 13:299–302. 6323346

[16] Waxman M Faegenburg D Waxman JS Janelli DE. Malignant fibrous histiocytoma of the colon associated with diverticulitis. Dis Colon Rectum. (1983) 26:339–43. doi: 10.1007/bf02561712, 6317305

[17] Spagnoli LG Dell'Isola C Sportelli G Mauriello A Rizzo F Casciani CU. Primary malignant fibrous histiocytoma of storiform-pleomorphic type: a case report of an ano-rectal localization. Tumori. (1984) 70:567–70. doi: 10.1177/030089168407000616, 6099619

[18] Baratz M Ostrzega N Michowitz M Messer G. Primary inflammatory malignant fibrous histiocytoma of the colon. Dis Colon Rectum. (1986) 29:462–5. doi: 10.1007/bf02561588, 3013530

[19] Satake T Matsuyama M. Cytologic features of ascites in malignant fibrous histiocytoma of the colon. Acta Pathol Jpn. (1988) 38:921–8. doi: 10.1111/j.1440-1827.1988.tb02363.x, 2847480

[20] Flood HD Salman AA. Malignant fibrous histiocytoma of the anal canal. report of a case and review of the literature. Dis Colon Rectum. (1989) 32:256–9. doi: 10.1007/bf02554541, 2537714

[21] Katz RN Waye JD Batzel EL Reiner MA Freed JS. Malignant fibrous histiocytoma of the gastrointestinal tract in a patient with neurofibromatosis. Am J Gastroenterol. (1990) 85:1527–30. 2173401

[22] Huang Z Wei K. Malignant fibrous histiocytoma of the ascending colon in a child. Am J Gastroenterol. (1993) 88:972–3. 8389096

[23] Murata I Makiyama K Miyazaki K Kawamoto AS Yoshida N Muta K . A case of inflammatory malignant fibrous histiocytoma of the colon. Gastroenterol Jpn. (1993) 28:554–63. doi: 10.1007/bf02776955, 7690726

[24] Makino M Kimura O Kaibara N. Radiation-induced malignant fibrous histiocytoma of the transverse colon: case report and review of the literature. J Gastroenterol. (1994) 29:767–71. doi: 10.1007/bf02349285, 7874274

[25] Hiraoka N Mukai M Suzuki M Mukal M Maeda K Nakajima K‐i . Malignant fibrous histiocytoma of the cecum: report of a case and review of the literature. Pathol Int. (1997) 47:718–24. doi: 10.1111/j.1440-1827.1997.tb04448.x, 9361108

[26] Kawashima H Ikeue S Takahashi Y Kashiyama M Hara T Yamazaki S . Primary malignant fibrous histiocytoma of the descending colon. Surg Today. (1997) 27:851–4. doi: 10.1007/bf02385277, 9306609

[27] Singh DR Aryya NC Sahi UP Shukla VK. Malignant fibrous histiocytoma of the rectum. Eur J Surg Oncol. (1999) 25:447–8. doi: 10.1053/ejso.1999.0677, 10419723

[28] Udaka T Suzuki Y Kimura H Miyashita K Suwaki T Yoshino T. Primary malignant fibrous histiocytoma of the ascending colon: report of a case. Surg Today. (1999) 29:160–4. doi: 10.1007/bf02482242, 10030742

[29] Okubo H Ozeki K Tanaka T Matsuo T Mochinaga N. Primary malignant fibrous histiocytoma of the ascending colon: report of a case. Surg Today. (2005) 35:323–7. doi: 10.1007/s00595-004-2915-1, 15815852

[30] Gupta C Malani AK. Primary malignant fibrous histiocytoma of the colon. Clin Gastroenterol Hepatol. (2006) 4:xxviii. doi: 10.1016/j.cgh.2006.01.004, 16630767

[31] Bosmans B de Graaf EJ Torenbeek R de Graaf EJR Tetteroo GWM. Malignant fibrous histiocytoma of the sigmoid: a case report and review of the literature. Int J Color Dis. (2007) 22:549–52. doi: 10.1007/s00384-006-0162-1, 16896996

[32] Fu DL Yang F Maskay A Long J Jin C Yu XJ . Primary intestinal malignant fibrous histiocytoma: two case reports. World J Gastroenterol. (2007) 13:1299–302. doi: 10.3748/wjg.v13.i8.1299, 17451221 PMC4147015

[33] Kim BG Chang IT Park JS Choi YS Kim GH Park ES . Transanal excision of a malignant fibrous histiocytoma of anal canal: a case report and literature review. World J Gastroenterol. (2008) 14:1459–62. doi: 10.3748/wjg.14.1459, 18322967 PMC2693701

[34] Azizi R Mahjoubi B Shayanfar N Anaraki F Zahedi-Shoolami L. Malignant fibrous histiocytoma of rectum: report of a case. Int J Surg Case Rep. (2011) 2:111–3. doi: 10.1016/j.ijscr.2011.01.013, 22096699 PMC3199686

[35] Wang YJ Tang SS Zhao Y. Contrast-enhanced sonographic appearance of malignant fibrous histiocytoma in the sigmoid colon: a case report. J Clin Ultrasound. (2012) 40:439–42. doi: 10.1002/jcu.20862, 22886378

[36] Ji W Zhong M You Y You YAN Hu KE Wu BIN. Primary malignant fibrous histiocytoma of the colon: a case report and review of the literature. Mol Clin Oncol. (2016) 4:1006–8. doi: 10.3892/mco.2016.849, 27284436 PMC4887843

[37] Kodera K Hoshino M Takahashi S Hidaka S Kogo M Hashizume R . Surgical management of primary undifferentiated pleomorphic sarcoma of the rectum: a case report and review of the literature. World J Surg Oncol. (2022) 20:199. doi: 10.1186/s12957-022-02671-6, 35698131 PMC9195365

[38] McCombs D Condon K Roybal J Warrier R Falcon C. Pediatric undifferentiated pleomorphic sarcoma of the cecum. Ochsner J. (2023) 23:77–81. doi: 10.31486/toj.22.0042, 36936487 PMC10016210

[39] Qureshi P Zeb S Tryggvason T Qureshi PAAA Þórisson A. Undifferentiated pleomorphic sarcoma of the descending colon: an infrequent occurrence. Cureus. (2024) 16:e61346. doi: 10.7759/cureus.61346, 38947723 PMC11214121

